# The biosynthetic pathway of 2-azahypoxanthine in fairy-ring forming fungus

**DOI:** 10.1038/srep39087

**Published:** 2016-12-19

**Authors:** Tomohiro Suzuki, Naoki Yamamoto, Jae-Hoon Choi, Tomoyuki Takano, Yohei Sasaki, Yurika Terashima, Akinobu Ito, Hideo Dohra, Hirofumi Hirai, Yukino Nakamura, Kentaro Yano, Hirokazu Kawagishi

**Affiliations:** 1 Center for Bioscience Research and Education, Utsunomiya University, 350 Mine-machi, Utsunomiya, Tochigi 321-8505, Japan; 2 Research Institute of Green Science and Technology, Shizuoka University, 836 Ohya, Suruga-ku, Shizuoka 422-8529, Japan; 3 Bioinformatics Laboratory, School of Agriculture, Meiji University, 1-1-1 Higashi-Mita, Kawasaki 214-8571, Japan; 4 Graduate School of Integrated Science and Technology, Shizuoka University, 836 Ohya, Suruga-ku, Shizuoka 422-8529, Japan; 5 Graduate School of Science and Technology, Shizuoka University, 836 Ohya, Suruga-ku, Shizuoka 422-8529, Japan

## Abstract

“Fairy rings” resulting from fungus-stimulated plant growth occur all over the world. In 2010, 2-azahypoxanthine (AHX) from a fungus *Lepista sordida* was identified as the “fairy” that stimulates plant growth. Furthermore, 2-aza-8-oxohypoxanthine (AOH) was isolated as a common metabolite of AHX in plants, and the endogenous existence of AHX and AOH in plants was proved. The structure of AHX allowed us to hypothesize that AHX was derived from 5-aminoimidazole-4-carboxamide ribonucleotide (AICAR). Thus, we performed a feeding experiment that supplied AICAR to *L. sordida*. Consumption of AICAR and accumulation of AHX were observed after feeding. The mycelia extract had enzymatic activity of *adenine*/*5-aminoimidazole-4-carboxamide phosphoribosyltransferase (APRT*). *APRT* gene of *L. sordida* revealed its structural characteristics in homology modeling and showed transcriptional enhancement after feeding. These results support that AHX was synthesized from AICAR and AHX biosynthesis was transcriptionally controlled by AICAR, indicating the presence of novel purine metabolic pathway in *L. sordida*.

“Fairy rings” is a disease symptom in lawn; rapidly growing, lush green circular bands of grass and/or circles of mushrooms are observed^1^. It has been found that more than 60 of basidiomycete fungi form fairy rings^1^,^2^. Fairy ring disease has been found on all grass types all over the world^3^, particularly on golf courses and athletic fields. It is often observed in hot, dry and drought weather conditions. The disease symptoms shows plant withering, wilting in rings, or activation of plant growth such as dark green turf grass^4^, leading to stimulated, or no influence on growth^5^.

*Lepista sordida* (division: Basidiomycota, order: Agaricales) has been a targeted fungus to study the fairy ring formation mechanism in research. Fairy rings caused by *L. sordida* have been reported on Zoysia grass (*Zoysia matrella and Zoysia japonica* Steud)^5^. Recently, insight into the molecular mechanism of fairy ring formation lead to the isolation of a plant growth regulator, 2-azahypoxanthine (AHX), from the fungus^6^. AHX stimulated the growth of various kinds of plants including turfgrass^6^. Subsequently, 2-aza-8-oxohypoxanthine (AOH) was isolated from rice as a derivative metabolite of AHX^7^. Both the compounds increased grain yields of wheat and rice in field experiments^8^. AHX is chemically synthesized from 5-aminoimidazole-4-carboxamide (AICA); AICA reacts with NaNO_2_ and then with NH_3_, giving AHX^7^. AHX and its ribotide (AICAR) are common members on the purine metabolic pathway in animals, plants and microorganisms, and AICAR is a precursor of fundamental metabolites such as inosine monophosphate (IMP), inosine, hypoxanthine, xanthine and uric acid in the pathway. From our findings and the facts mentioned above, we hypothesized that plants themselves produce AHX through a pathway similar to the chemical synthesis, and we proved the hypothesis experimentally^7^. However, it remains unknown whether the pathway exists in fairy-ring forming fungi and how AICA is synthesized in cells.

Adenine/5-aminoimidazole-4-carboxamide phosphoribosyltransferase (APRT; EC 2.4.2.7) also called as nucleotide pyrophosphorylase is involved in AHX biosynthesis. APRT catalyzes the reversible transfer of the 5′-phosphoribosyl moiety from α-D-5-phosphoribosyl-1-pyrophosphate (PRPP) to adenine to form adenosine-5-monophosphate (AMP) and pyrophosphate (PPi)^9^. It also recognizes hypoxanthine, 2,6-diaminopurine, and AICA as substrates, although its binding affinities to the substrates are weaker compared to adenine^10^. Nucleotide pyrophosphorylase purified from beef liver converted AICAR and pyrophosphate to AICA and 5-phosphoribosyl pyrophosphate^11^. *APRT* is conserved in various organisms: animals, bacteria, and plant species^12^,^13^,^14^,^15^,^16^,^17^. The primary role of APRT is in adenine salvage and recycling^18^,^19^. In APRT-deficient human cells, adenine is converted to 2,8-dihydroxyadenine (DHA) by xanthine dehydrogenase, and accumulation of DHA is toxic to renal tubular and interstitial cells^20^. In the flowering plant, Arabidopsis, mutations in *APRT* affected pollen development causing male sterile^21^. APRT protein and its crystal structure from animals, bacteria, protista, fungi and archaea have been determined until now has been well-studied^9^,^22^,^23^,^24^,^25^,^26^,^27^. By contrast, there is still no knowledge on APRT in fairy ring-forming fungi.

The goal of this study is to examine whether AICAR could be a substrate for AHX biosynthesis in *L. sordida*. In this study, we provide answers for the three following points: 1) how feeding of AICAR in *L. sordida* mycelia culture affects on AHX accumulation and APRT gene expression, 2) what are the structural features of APRT, and 3) whether *L. sordida* mycelia has an enzyme activity for interconversion between AICAR and AICA or not.

Here, we performed a feeding experiment that supplied AICAR into *L. sordida*, determination of AICAR-converting activity of the crude enzyme extract from the mycelia, identification and isolation of an *APRT* gene in the fungus, and investigation of expression levels of the *APRT* gene during the feeding experiment.

## Results

### Feeding of AICAR into mycelia culture

**Figure 1:**
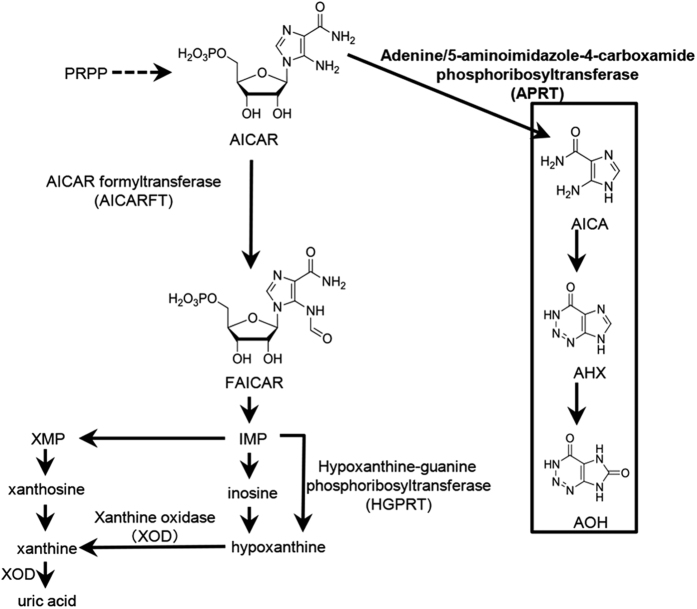
Hypothetical biosynthetic pathway for AHX/AOH. PRPP, phosphoribosyl pyrophosphate; FAICAR, *N*-formyl-5-aminoimidazole-4-carboxamide; IMP, inosine monophosphate; XMP, xanthosine monophosphate. Dotted arrow indicates multiple enzymatic reaction steps.

**Figure 2:**
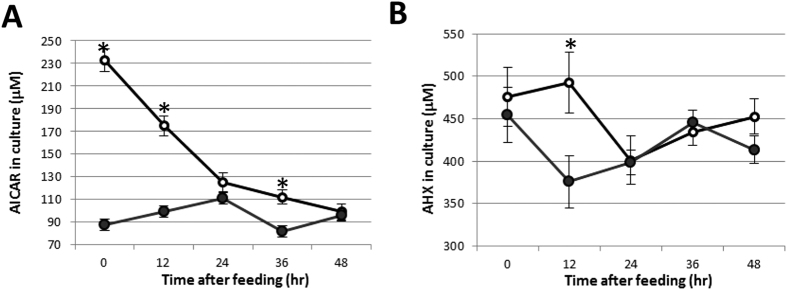
Transitional changes of AICAR and AHX contents in mycelia culture after feeding of AICAR. Error bar represents standard deviation of five biological replicates. Asterisk represents statistical significance of difference from control at 5% level. (**A**) AICAR. (**B**) AHX. The sample after feeding of AICAR is indicated with white circles and control (without AICAR) is indicated with black circles.

In the hypothesized AHX/AOH biosynthetic pathway, AICAR is the initial precursor through AICA (Fig. 1). If this hypothesis is true, provision of AICAR to *L. sordida* should activate accumulation of AHX in the fungus. Therefore, we fed AICAR to *L. sordida* culture and quantified the amounts of AICAR and AHX until 48 h after feeding. AICAR concentration of non-fed control was between 81.5 ± 7.8 and 111.0 ± 11.5 μM during the experiment. The feeding increased the concentration of AICAR to 232.8 ± 9.8 μM from 87.8 ± 12.1 μM at 0 h, and then continuously decreased until 48 h after feeding (Fig. 2A). In comparison of these two samples, significant difference was observed at 12 h after feeding (Fig. 2A). These results indicated that a large part of AICAR fed was consumed within 24 h after feeding. However, no significant difference was observed in the non-fed control of AHX, its concentration decreased to 375.8 ± 30.8 μM from 454.5 ± 32.8 μM at 12 h after feeding. The feeding of AICAR slightly increased AHX accumulation to 475.9 ± 35.2 μM at 12 h after feeding, and decreased to 401.3 ± 28.1 μM at 24 h after feeding. Comparing these two results show significant difference between fed and non-fed groups at 12 h after feeding (P ≤ 0.05 at 12 h). All the results indicated that there exists a correlation between consumption of AICAR and accumulation of AHX in the mycelia culture at 12 h after feeding and AICAR was utilized for AHX biosynthesis in the mycelia.

### Enzymatic activity assay in mycelia

**Figure 3:**
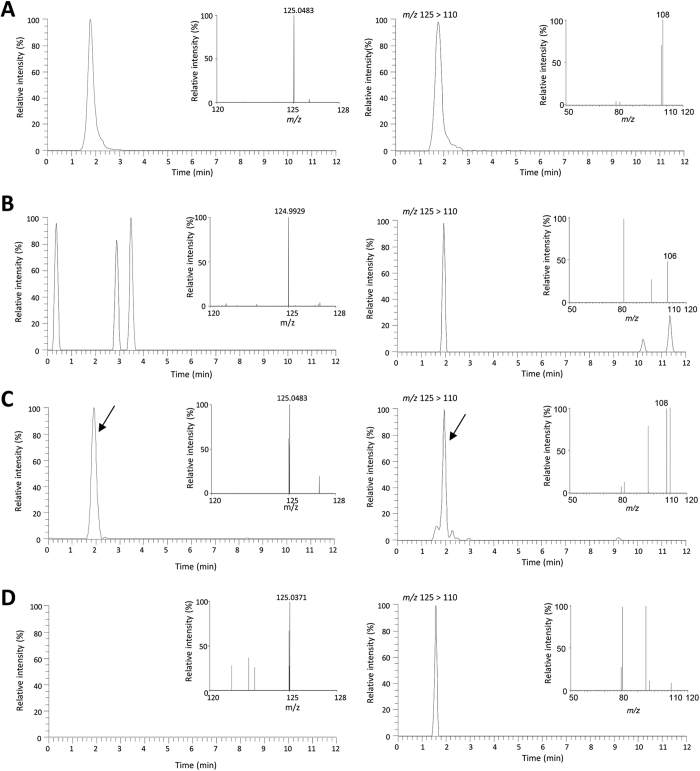
Analysis of APRT activity in crude enzyme extract by LC-MS/MS. Multiple reaction monitoring (MRM) for AICA including MS (left side) and MS/MS spectra (right side). (**A**) AICA standard. (**B**) Buffer (100 mM Tris-HCl, pH 8.3) and AICAR. (**C**) Crude enzyme extract and AICAR. (**D**) Crude enzyme extract and distilled water. Arrow indicates a signal of AICA.

Crude extract from the mycelia culture was prepared and tested for AICAR conversion to AICA in the extract, using LC-MS/MS. As shown in Fig. 3, the crude extract with AICAR produced AICA. Whereas, AICA was not detected in reactions without either substrate or enzyme (Fig. 3B and D). These results showed that mycelia contained enzyme(s) converts AICAR to AICA.

### Identification of *APRT* gene

To explore *APRT* genes in *L. sordida*, genomic scaffolds were generated by next-generation sequencing approach. A hybrid sequencing strategy by 454 pyrosequencing and Illumina sequencing techniques was employed. For 454 pyrosequencing, 915,457 of reads were generated. For Illumina sequencing, 66,506,146 and 71,023,220 short reads were generated from the PE-library and MP-library, respectively. The short reads were preprocessed to extract 79,179,992 of high-quality reads in total (See details in Materials and Methods and Supplementary Table 1). The data were conducted to a hybrid assembly strategy (See details in Materials and Methods). As a result, 812 of genomic scaffolds were constructed (Supplementary Table 2). The scaffold sequences were then applied to a search for *APRT* gene. Namely, we queried an APRT sequence in yeast (Genbank ID: L14434.1) by nhmmer search against the genomic scaffolds. The search identified a genomic scaffold including a potential *APRT* gene in *L. sordida* as a top hit with statistical significance (E-value of 8E-4) (Supplementary Fig. 1A). The second hit showed no statistical significance (E-value of 5.3). By aligning the APRT gene sequence with those of *Coprinopsis cinerea* and *Moniliophthora roreri* (Genbank ID: XM_001837049 and XM_007844891, respectively), the coding region was predicted (Supplementary Fig. 1B).

### cDNA cloning of *APRT* in *L. sordida*

**Figure 4:**
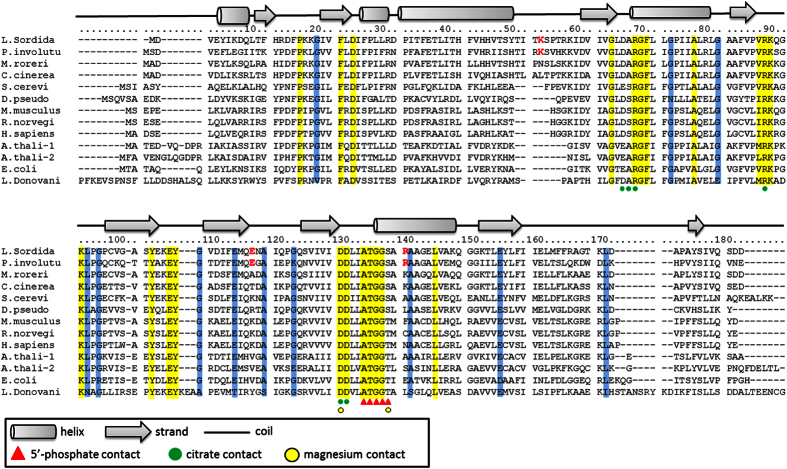
A multiple alignment of APRTs in 13 species. Conserved sequence in all the APRT and 12 APRT are colored in yellow and blue, respectively. Residues involved in 5′-phosphate binding and Mg-pyrophosphate binding were circled in red and in black, respectively. The following APRTs were applied (GenBank accession numbers in parentheses): P.involutu, *Paxillus involutus* (KIJ20178.1); M.roreri, *Moniliophthora roreri* (XM_007844891.1); C. cinerea, *Coprinopsis cinerea* (XM_001837049.1); S. cerevi, *Saccharomyces cerevisiae* (P49435); D. pseudo, *Drosophila pseudoobscura* (L06281.1); M. musculus, *Mus musculus* (NM_009698.2); R. norvegi, *Rattus norvegicus* (NM_001013061.1); H. sapiens, *Homo sapiens* (P07741); A. thali-1, *Arabidopsis thaliana* (L19637.1); A. thali-2, *Arabidopsis thaliana* (X96867.1); E. coli, *Escherichia coli* (M14040.1); L. Donovani, *Leishmania Donovani*; (1QB7_A).

cDNA of *APRT* was isolated from the mycelia of *L. sordida*. The cDNA amplified by RT-PCR was cloned into a vector pMD20-T sequenced (deposited to DDBJ, accession number LC060066). cDNA sequence perfectly matched with the predicted coding sequence from the genomic scaffold (data not shown). The open reading frame was 546 bp length, and it coded 182 amino acid protein (Supplementary Fig. 2). The deduced amino acid sequence showed significant homologies with those of APRTs in *Saccharomyces cerevisiae* (51%), *Drosophila pseudoobscura* (43%), *Mus musculus* (44%), *Rattus norvegicus* (41%), *Homo sapiens* (40%), *Arabidopsis thaliana* (44% and 38%), *Escherichia coli* (40%), and *Leishmania donovani* (32%).^9^,^14^,^15^,^16^,^23^,^28^,^29^,^30^,^31^. We observed core motif sequences of APRT in the *L. sordida* APRT (Fig. 4). Phosphoribosyl pyrophosphate (PRPP)-binding motifs, composed of citrate contact region in crystal structures of *L. donovani* APRT^23^ and 5′-phosphate contact region in *S. cerevisiae* APRT^9^, were completely conserved in *L. sordida*. The amino acid position of 129 and 137, residues of Mg^2+^ binding were also conserved in APRTs^31^,^32^,^33^,^34^,^35^,^36^.

In structural analysis of the APRT, predicted five of α-helix and nine of β-strand structures seen in *S. cerevisiae* were also observed in the APRT of *L. sordida* (Fig. 4). A missing region (51-Thr-Ile-Thr-Lys-54), which was conserved among fungi, was also present. This region formed a part of loop in homology modeling with APRT in *S. cerevisiae* by SWISS-MODEL (Supplementary Fig. 3). The insertion of the missing region in APRT in *L. sordida* provided a larger space inside of the loop. Notably, the missing region contained a distinctive conserved amino acid Lys-54 between *L. sordida* and a fairy ring-forming fungi *Paxillus involutus* (Fig. 4). ProtScale analysis represented the loop region was more hydrophilic than that of *S. cerevisiae* (Supplementary Fig. 4). These results suggested that the loop in *L. sordida* has high flexibility.

### Gene expression analysis of *APRT* and *AICARFT* gene

**Figure 5:**
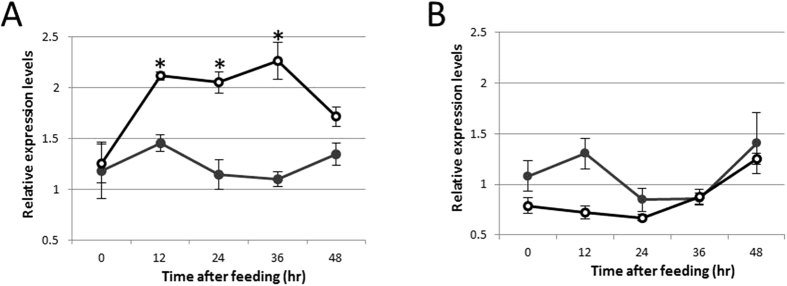
Gene expression of *APRT* and *AICARFT* in response to AICAR feeding. X-axis represents normalized gene expression level of the *APRT* (**A**) and *AICARFT* (**B**) by an actin. Error bar indicates standard deviation of four biological replicates. Asterisk represents significant difference to from negative control (P < 0.05 by Student’s T-test). The sample after feeding of AICAR is indicated with white circles and control (without AICAR) is indicated with black circles.

We examined the effect of AICAR feeding on *APRT* and *AICARFT* at mRNA level. Gene expression of *APRT* and *AICARFT* in mycelia culture was analyzed by qRT-PCR. In gene expression of *APRT*, the relative expression level of control did not change significantly. In contrast, APRT started to increase at 12 h after feeding and maintained at high levels until 36 h after feeding (Fig. 5A). The response was consistent with AHX accumulation at 12 h after feeding (Fig. 2B). The *AICARFT* expression level of non-fed control was between 0.84 ± 0.05 and 1.4 ± 0.3 during the experiment. Although there was no significant difference of AICARFT expression level between fed and non-fed groups, expression level of AICAR fed groups tended to slightly decrease at 12 h after feeding. These results suggested that *AICARFT* constantly expressed and the significant response to AICAR feeding was not observed in *L. sordida* mycelia culture.

## Discussion

In this study, we established a feeding experiment procedure in *L. sordida* mycelia culture for exploring enzyme genes in AHX and AOH biosynthetic pathway. After feeding of AICAR to the mycelia culture, consumption of AICAR and accumulation of AHX were observed in the culture (Fig. 2). In the feeding experiment, the *APRT* gene enhanced after 12 h of feeding (Fig. 5). These results suggested that the *APRT* gene acted upon AHX and AOH biosynthesis. Moreover, the presence of AICAR-converting enzyme in the crude enzyme extract of *L. sordida* mycelia was demonstrated by detecting AICA in LC-MS analysis. Our data suggested that the *APRT* gene responded to addition of AICAR and also might be involved in converting AICAR to AICA in the mycelia extract.

Despite the drastic reduction of AICAR until 48 h after feeding, clear increase of AHX was only observed at 12 h after feeding. But, gene expression level of *APRT* was high for more than 36 h which indicated that synthesized AHX might be promptly metabolized or converted to other derivative(s). Alternatively, AICAR might have been utilized for biosynthesis of more fundamental metabolites like IMP, inosine, hypoxanthine, uric acid and so on. In fact, AICAR formyltransferase (AICARFT), which catalyzes transfer of a formyl group to AICAR to produce *N*-formyl-5-aminoimidazole-4-carboxamide (FAICAR), was coded in the genome assembly of *L. sordida*^37^. Although AICARFT consumes AICAR and biosynthesizes the downstream metabolites, our data suggested that significant changes in expression level of *AICARFT* gene after AICAR feeding were not observed. The reason for this disagreement between results of feeding experiment and gene expression analysis in this study still remains unknown. Further analysis is needed to disclose other unknown metabolite(s) and derivative(s) in the future.

The consistent relationship between AICAR feeding and response of *APRT* at 12 h after feeding was observed in this report. Hence, what’s upstream of the APRT in *L. sordida*? To our knowledge, there is no report for transcriptional regulation mechanism of APRT in mushrooms. Mummaneni *et al*. (1993) reported that the promoter region of a mouse APRT was subjected to DNA methylation^38^. In the Chinese hamster, two putative GC factor (GCF) -binding sites suppress transcription of APRT^39^. In order to test a potential involvement of GCF for AHX biosynthesis and fairy ring formation, the feeding experiment of AICAR and the genome sequence in *L. sordida* would be useful. Detailed genome annotation of *L. sordida* is now underway to clarify further understanding of fairy ring formation.

Fungal genomes were analyzed by next-generation sequencing^40^. As fairy ring forming fungi, only *P. involutus* was sequenced^41^. However, there is no mechanistic information on the fairy ring formation in the species. Here, to promote elucidation of molecules for fairy ring formation mechanism, we conducted genome sequencing in *L. sordida* as its fairy ring formation mechanism was previously reported^6^. Hybrid assembly of the generated sequence reads provided a reference genome scaffold in *L. sordida*, and it was sufficient for identification of the *APRT* gene. The comparison of APRT sequences between *L. sordida* and *P. involutus* revealed conserved amino acid residues between fairy ring-forming fungi. The genomic information of *L. sordida* and *P. involutus* opens new avenues for elucidation of the mechanism of fairy ring formation.

**Figure 6:**
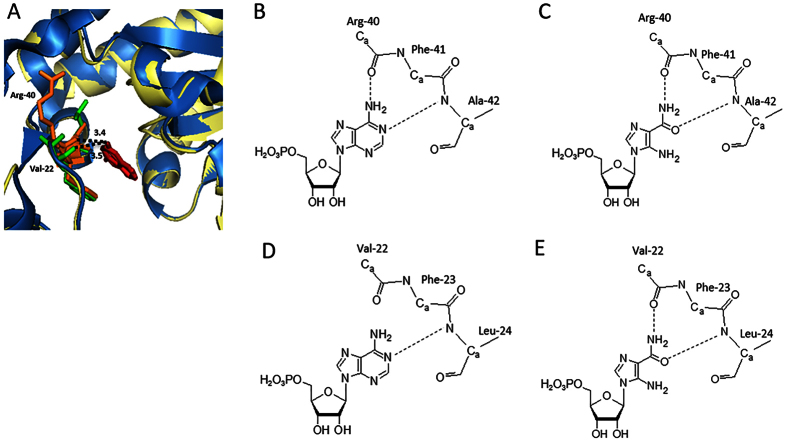
Structure comparison between *L. sordida* and *L. donovani* APRT. (**A**) Three-dimensional structural modeling was performed on SwissModel server with the *L. donovani* (PDB code, 1QB7) as the template. Ribbon diagram show the overlap of the *L. donovani* APRT monomer (blue) and *L. sordida* APRT monomer (yellow). Adenine, amino acid residues involved in the adenine binding of *L. sordida* (22-Val-Phe-Leu-24) and *L. donovani* (40-Arg-Phe-Ala-42) APRT are shown in red, green and orange, respectively. Distance between adenine and the carbonyl group is in angstroms. Schematic representation of putative adenine binding site of *L. donovani* APRT contacts to AMP (**B**) and AICAR (**C**). Schematic representation of putative adenine binding site of *L. sordida* APRT contacts to AMP (**D**) and AICAR (**E**).

A crystal structure analysis revealed the adenine binding domain of APRT in *L. donovani*^23^. This study reported Arg-40, Phe-41, and Ala-42 (corresponding to Val-22, Phe-23, and Leu-24, respectively, in *L. sordida*, Fig. 4) were residues to form hydrogen bonds with adenine of AMP. Interestingly, Sarver and Wang (2002) documented that a point mutation of the Phe of APRT in *Giardia lamblia* to Trp residue showed no obvious effect on adenine binding property^42^. Furthermore, the remaining two residues, Arg-40, and Ala-42 were substituted to Val and Leu, respectively, in mushroom-forming fungi (Fig. 4) and the substitutions caused a conformational change in region in our homology modeling (Fig. 6A). In *L. sordida*, the position of the Val residue shifted to just below Arg-40 of *L. donovani* APRT, and it extended the distance between adenine and the carbonyl group of the Val for the hydrogen bond (Fig. 6A and B). We propose the binding model of AICAR and AMP to *L. donovani* and *L. sordida* APRT as shown in Fig. 6B–D and E. The result suggested that *L. sordida* APRT has lower affinity to AMP, and might cause an increase in relative affinity to AICAR. Thus, decrease in affinity for AMP might affect the efficiency of AICAR usage in APRT from *L. sordida*.

Several other sites showing diversity between fairy ring-forming fungi and other organisms were found (Fig. 4). Although the position of 116 was neutral and acidic amino acid in other APRTs, Glu-116 was conserved between *L. sordida* and *P. involutes* (Fig. 4). Amino acid positioned on the dimer surface (due to electrical charge) might be involved resulting in their formation and the stability (Supplementary Fig. 3C). The position 139 was also substituted with Arg in fairy ring-forming fungi, and Arg-139 is component of α-helix near 5′ phosphate binding motif, 133-Ala-Thr-Gly-Gly-Ser-137 (Supplementary Fig. 3D). Although the side chain of Arg-139 was not facing the direction of the active site, its strong basicity might affect the conformation of active site. In the amino acid positions of 12, 34, 48, 53, 100, 165 and 167, residues were not conserved between fairy-ring forming fungi and other organisms (Fig. 4). In fairy ring-forming fungi, the position 53 in the missing region was substituted with a polar amino acid (Thr-53 in *L. sordida* or Arg-53 in *P. involutes*). The amino acid positioned at residue 165 was located above the active-site and might be involved in the attraction of the substrate.

In conclusion, we provided evidence of AHX biosynthetic pathway in *L. sordida*; 1) feeding of AICAR in *L. sordida* mycelia culture increased AHX accumulation and gene expression level of APRT, 2) APRTs among mushroom-forming fungi had evolutionarily conserved domains in active site, 3) *L. sordida* mycelia had an enzyme activity for conversion of AICAR to AICA. It will take some time to mine all genes involved in the biosynthesis of AHX and AOH. However, this study disclosed further metabolism of AICA which is a common member of the purine metabolic pathway in animals, plants, and microorganisms, whose metabolism had remained elusive.

## Methods

### Strain and culture condition

*Lepista sordida* provided from Nasu Biofarm, Ltd. (Japan) was pre-cultured in PYG medium (0.3% polypepton, 0.3% yeast extract, 1% glucose) supplemented with 1.8% of agar to obtain actively growing mycelia. The inoculated mycelia were incubated at 25 °C for 3 weeks under dark condition. After growth, the strain on the medium was stored at 4 °C. The mycelia were used within two months for following experiments below.

For preparation of genomic DNA, the strain was grown in YG medium (1% glucose, 0.3% yeast extract, 3.7 mM KH_2_PO_4_ and 3.5 mM Na_2_HPO_4_) in a flask with 120 rpm in dark condition. The culture was performed at 25 °C for 2 weeks. The mycelia were collected by filtration through 0.2 μm-membrane filter (Advantec Toyo Co., Japan).

For the feeding experiment and gene expression analysis, 8.5-mm-diameter of gel disks were punched out from the growing edge of mycelium. Two disks were each placed into a 50 mL Erlenmeyer flask containing 10 mL of YG medium. After incubation of the flasks at 25 °C for 12 days, 100 μL of 10 mM AICAR was added to the cultures, and then further incubated for 12, 24, 36, and 48 h. Cultured mycelia was prepared with five biological replicates.

### RP-HPLC analysis

Amounts of AICAR and AHX in mycelia culture were determined by reversed-phase high-performance liquid chromatography (RP-HPLC). After feeding of AICAR to mycelia culture, 20 mL of methanol and 100 μL of 10 mM allopurinol (internal standard, final concentration 100 μM) were added to all of the samples. Then the cultures were homogenized by Polytron PT 1200E homogenizer (Kinematica, Switzerland) for 1 min. The cell suspensions were extracted with 20 mL of MeOH and filtrated. Each MeOH extract was dried at 35 °C by a rotary evaporator and re-dissolved in 10 mL of 10 mM ammonium formate buffer (pH 4.0), and then subjected to RP-HPLC. RP-HPLC was carried out to quantify AICAR and AHX under the following conditions: a PU-2089 gradient pump, AS-2010 multi wavelength detector, AS-2055 plus autosampler (JASCO Co., Japan); column, CAPCELL PAK ADME (4.6 mm × 250 mm; Shiseido Co., Japan); mobile phase, solution A: acetonitrile, solution B: 10 mM ammonium formate buffer (pH 4.0), 0–10 min, 2% Solution A; 10–40 min, 2–50% sol. A; flow rate,1 mL/min; and UV wavelength, 250 nm.

### Detection of AICA by LC-MS/MS Analysis

AICAR-converting activity to AICA in crude enzyme extract of the mycelia was tested by LC-MS/MS analysis. The mycelia were frozen in liquid nitrogen and ground to a powder with a motor and pestle. The mycelia powder was extracted with extraction buffer containing 100 mM Tris-HCl (pH 8.3), 0.2% (w/v) 3-(3-cholamidepropyl) dimethylammonio-1-propanesulphonate, 10% (v/v) glycerol, 1 mM phenylmethylsulfonyl fluoride and 20 mM 2- mercaptoethanol at 4 °C for 3 h with shaking. The extract was centrifuged at 10,000 g for 20 min, and the supernatant was desalted by Centrifugal ultrafiltration using Amicon Ultra Centrifugal Filter Devices (3,000-Da cutoff; MerkMillipore, Japan). Ultrafiltration was repeated 3 times by adding fresh 100 mM Tris–HCl buffer (pH 8.3). After adding AICAR (final concentration 0.2 mM) to desalted crude extract, the sample was incubated at 28 °C for 8 h and subjected to LC-MS/MS analysis. AICA in the sample was detected on LC-MS/MS by comparing the retention time and precursor ion with those of authentic one. Analyses were performed with a Shimadzu UPLC system (Shimadzu, Japan) coupled to an LTQ Orbitrap mass spectrometer (Thermo Fisher Scientific, Waltham, MA, USA) equipped with an electrospray ionization probe. A PC-HILIC column (ϕ 2 × 100 mm, 3 μm; Shiseido, Japan) was used in the analysis (injection volume, 10 μL; solvent, 95% MeOH with 0.05% formic acid; flow rate; 0.2 mL/min). After each analysis, the column was re-equilibrated for 10 min at the initial conditions prior to the next sample analysis. MS analysis was performed in the negative FTMS mode at a resolution of 30,000 at *m*/*z* 400 with the following source parameters: sheath gas flow, 50; auxiliary gas flow rate, 10; tube lens, −63 V; capillary voltage, −16 V; ion spray voltage, 3 kV.

### DNA preparation

The mycelia were frozen in liquid nitrogen and powdered with a motor and pestle. The powdered sample was used for extracting genomic DNA by using DNeasy Plant Mini Kit (Qiagen, Germany) following the instruction manual. Obtained DNA was quantified by using PicoGreen dsDNA Quantification Reagent (Invitrogen, USA) and analyzed on 0.7% of agarose electrophoresis to check the quality. The collected genomic DNA was used for genomic library construction.

### Sequencing by Illumina/GAIIx

Two genomic DNA libraries for paired-end (PE) sequencing and mate paired-end (MP) sequencing were constructed by using Truseq DNA sample preparation kit and Mate pair library sample preparation kit, respectively, following the instruction manuals (Illumina, USA). For PE sequencing, 1 μg of genomic DNA was fragmented and electrophoresized on 2% of agarose to collect a DNA fraction of approx. 300 bp length. For MP sequencing, 10 μg of genomic DNA was fragmented and conducted to end-repaired. Subsequently, the 3′ ends of the DNA fragments were biotinylated by incorporating labeled dNTPs and electrophoresized on 0.6% of certified megabase agarose gel (Bio-Rad Laboratories Inc, USA) to collect a DNA fraction of approx. 3 kb length. DNA fragments in the fraction were circulated and then fragmented into 350–650 bp length. The biotinylated DNA was purified by using Dynabeads M-280 Streptavidin Magnetic Beads (Life Technologies, USA).

Each genomic DNA library was made blunt ended with End Repair Enzyme (Illumina) in the presence of 2.5 mM dNTPs and 10 mM ATP. Adenine nucleotide was added to the 3′ ends of the blunt ended cDNA with Klenow fragment (3′ to 5′ exominus) in the presence of 1 mM dATP by incubating at 37 °C for 30 minutes. The DNA with adenine on its ends was ligated with adapters provided by Illumina (PE; TruSeq Universal Adapter and TruSeq Indexed Adapter, MP; paired end (PE) oligo adapters) using T4 DNA ligase at room temperature for 15 min. The DNA was amplified with two adapter primers (Illumina) (PE; TruSeq Universal Adapter primer, and IlluminaTruSeq Adapter primer, MP; PE primers 1.0 and 2.0) with initial denaturing step at 98 °C for 30 s, followed by 15 cycles at 98 °C for 10 s, 65 °C for 30 s, 72 °C for 30 s with a final extension cycle at 72 °C for 5 min. The PCR products were purified with Qiaquick PCR purification kit and gel-extracted.

Whole genome sequencing using Illumina Genome Analyzer GAIIx (Illumina) was carried out by the method of 100 bp of paired-end. One lane for each library was used.

### Sequencing by Roche/GS FLX Titanium

A paired-end genomic DNA library 8 K and a fragment genomic DNA library were constructed to be sequenced by GS FLX Titanium in Dragon Genomics Center (Takara Bio Inc., Japan). Construction of the paired-end library was conducted following the method described in GS FLX Paired End DNA Library Preparation Method Manual (provided from Roche Diagnostics K. K. Japan). Ten μg of the genomic DNA was mechanically digested by HydroShear (Digilab, USA) into approx. 8 kb of DNA fragments. The both termini of the DNAs were blunted and ligated with an adaptor with biotinated loxP site for cyclizing them. The DNA fraction was separated by agarose electrophoresis, and DNA fragments of 6.5 to 9.5 kb were eluted from the agarose gel by using the Elutrap Electroelution System (GE Healthcare, Japan). Obtained DNAs were circulated by a reaction of Cre Recombinase. The DNAs were then conducted to fragmentation by an Acoustic Solubilizer (Covaris, USA) and blunting the termini. After binding the DNAs to streptavidin-coupled dynabeads by using the affinity with biotin, the collected DNAs were ligated with the Library Adapters provided by the manufacture (Roche Diagnostics K. K.). The DNAs were amplified by PCR at 20 cycles by using a biotinated Amplification Primers (Roche Diagnostics K. K.) to purify the biotinated PCR products. Pool of denatured PCR products in alkaline treatment was designated as the paired-end library. For the fragment genomic DNA library, GS FLX Titanium Rapid Library Preparation Kit (Roche Diagnostics K. K.) was used following the manufacture’s manual. Briefly, one μg of the genomic DNAs was fragmentized by using Covaris S-series (Covaris, Inc., USA) and then ligated with RL adaptor included in GS FLX Titanium Rapid Library Preparation kit (Roche Diagnostics K. K.). From the DNA fraction, short DNAs were excluded by using Agencourt AMPure XP (Beckman Coulter, Inc., Brea, USA), and the resultant was designated as the sequence library.

Each sequence library was conducted to emulsion PCR after binding with capture beads. The capture beads were applied to sequencing by Genome Sequencer FLX + System.

### Preprocessing of sequence reads

Raw sequence data of the Illumina sequence data were subjected to trimming and quality filtering were done with an in house Perl script. Low-quality bases at the sequence read ends with the quality value < 10 in fastq files were trimmed. Low-quality reads with either of the following criteria, read length < 20 bp, average of quality score per read < 17, number of low-quality bases (quality value < 10) per read > 10% or N’s per read  > 0%, were removed.

Raw sequence data of the GS FLX sequence were processed by using the sff_extract software 0.3.0 (http://bioinf.comav.upv.es/sff_extract/index.html) to trim the adapter sequences.

### *De novo* assembly

Sequence reads derived from GAIIx and GS FLX Titanium were conducted to hybrid assembly by multi-step procedures. The sequence reads from GS FLX Titanium were assembled into contigs by Newbler^43^, and then the contigs were further integrated with Illumina mate pair short reads to generate genomic scaffolds by SSPACE^44^. At final, Illumina pair end reads were used to fill an indefinite nucleotide “N” in the genomic scaffolds by Gap closer^45^.

### Sequence search

HMMER (ver. 3.1) was applied to search an APRT gene sequence in *L. sordida*^46^. Query sequence was conducted to nhmmer search against the whole genome assembly in *L. sordida* with the default setting.

### cDNA cloning of APRT in *L. sordida*

RT-PCR was performed with PrimeScript^TM^ RT-PCR kit (TaKaRa Bio Inc., Japan). Total RNA was prepared by using RNeasy plant mini kit (Qiagen) and applied to 1st-stranded cDNA synthesis with oligo (dT) primer and a PrimeScript RT reagent kit (TaKaRa Bio Inc.). A pair of primers (5′-ATGGACGTTGAGTACATTAAAG-3′ and 5′-TCAATCATCCGATTGAACGATC-3′) were designed based on predicted nucleotide sequence of *APRT* gene and employed for PCR. Amplified PCR product was purified and cloned in a vector pMD20-T (TaKaRa Bio Inc.) using a Mighty TA cloning kit (TaKaRa Bio Inc.).

### Structural analysis of APRT protein

Multiple alignment of APRT was prepared using the ClustalX software (Thompson *et al*., 1997). The secondary structure of the *L. sordida* APRT was predicted by PSIPRED v3.3 on the PSIPRED server^47^. We predicted a protein structure of APRT in *L. sordida* using the Swiss-Model automated protein structure homology-modeling server with the template of the APRT in *Saccharomyces cerevisiae* (Protein data bank code, 1G2Q)^48^. Individual hydrophobicity was determined using the Protscale software at the EXPASY server (Gasteiger *et al*. 2005), with the amino acids scale of “Hphob/Kyte and Doolittle”^49^. Distance between adenine and the carbonyl group in the homology model was calculated with Pymol (https://www.pymol.org/).

### Quantitative gene expression analysis

Gene expression analysis was carried out using quantitative real-time RT-PCR (qRT-RT-PCR). qRT-RT-PCR was performed in 96-well plates with a LightCycler 480 real-time PCR instrument (Roche Diagnostics K. K.). One microgram of total RNA samples was incubated with 1 U deoxyribonuclease I (Invitrogen, USA) for 15 min at room temperature to remove genomic DNA, and EDTA was added to a final concentration of 2 mM to stop the reaction. After that, 1st-strand cDNAs were synthesized from total RNAs using a PrimeScript RT reagent kit (TaKaRa Bio Inc.) with oligo (dT) primer. Each 20 μL reaction mixture containing 50 ng of total cDNA, 10 μL FastStart Essential DNA Green Master (Roche Diagnostics K. K.), and 0.5 μM of each primer. Cycling conditions was set as follows: pre-incubation, 1 cycle of 95 °C for 10 min; amplification, 45 cycles of 95 °C for 10 s, 57 °C for 10 s and 72 °C for 10 s. The following primers were used: *APRT* forward primer, 5′-CTCCTCGGTCCAATAATCGC-3′; *APRT* reverse primer, 5′-AAATATGTCCACACCGTATTC-3′; *AICARFT* forward primer, 5′-CAAGATTGACGCTAAGCTCTTCGAG-3′; *AICARFT* reverse primer, 5′- GTGGCGACGATTAAGTCTGTAATTG. Gene expression levels were normalized by gene expression of an actin; primers 5′-CGTTGTTTGCGGTCG-3′ and 5′-GCTAAAATATCTTTG-3′ were designed.

## Additional Information

**How to cite this article**: Suzuki, T. *et al*. The biosynthetic pathway of 2-azahypoxanthine in fairy-ring forming fungus. *Sci. Rep.* **6**, 39087; doi: 10.1038/srep39087 (2016).

**Publisher's note:** Springer Nature remains neutral with regard to jurisdictional claims in published maps and institutional affiliations.

## Supplementary Material

Supplementary Information
