# Supplementary material for: The biosynthetic pathway of 2-azahypoxanthine in fairy-ring forming fungus
Source: Sci Rep. 2016 Dec 19;6:39087. doi: 10.1038/srep39087 (PMC5171910; doi:10.1038/srep39087)
Supplement: Supplementary Information [file srep39087-s1.pdf]

The biosynthetic pathway of 2-azahypoxanthine in fairy-ring forming fungus

Tomohiro Suzuki[1,2], Naoki Yamamoto[3,6], Jae-Hoon Choi[2,4], Tomoyuki Takano[3], Yohei Sasaki[3], Yurika Terashima[4], Akinobu Ito[4], Hideo Dohra[2], Hirofumi Hirai[2,4,5], Yukino Nakamura[3], Kentaro Yano[3], Hirokazu Kawagishi[2,4,5]

[1] Center for Bioscience Research and Education, Utsunomiya University, 350 Mine-machi, Utsunomiya, Tochigi 321-8505, Japan
[2] Research Institute of Green Science and Technology, Shizuoka University, 836 Ohya, Suruga-ku, Shizuoka 422-8529, Japan
[3] Bioinformatics Laboratory, School of Agriculture, Meiji University, 1-1-1 Higashi-Mita, Kawasaki 214-8571, Japan
[4] Graduate School of Integrated Science and Technology, Shizuoka University, 836 Ohya, Suruga-ku, Shizuoka 422-8529, Japan
[5] Graduate School of Science and Technology, Shizuoka University, 836 Ohya, Suruga-ku, Shizuoka 422-8529, Japan
[6] Present address: Plant Breeding, Genetics and Biotechnology Division, International Rice Research Institute, Metro Manila, Philippines

Supplementary table 1
Main features of sequence data.

	FLX+	GAIIx
Number of raw reads	915,457	137,529,366
Length of reads (bp)	376,450,318	13,890,465,966
Av. of length of reads (bp)	411	101
Number of high-quality reads		
Number of reads	915,457	79,179,992
Length of reads (bp)	376,450,318	7,514,231,108
Av. of length of reads (bp)	411	94.9

Supplementary table 2
Assembly summary.

Number of scaffolds	812
N50 (bp)	178878
Total size of scaffolds (bp)	36644714
Maximum scaffold length (bp)	899431
Minimum scaffold length (bp)	1999
Average scaffold length (bp)	45129

```
yeast APRT(L14434.1)            458   agttcgccaaagaaaagatcgactttattgcaggtatagaagctagaggccttctatttgggcca 522
                                      agt   cca    aaagatcga  ttat g  ggt t ga gc  g gg  ttct  t gg cca
potential L.sordida APRT      28662   AGTCACCCACGAGAAAGATCGATGTTATCGTTGGTCTTGACGCACGCGGTTTTCTCCTCGGTCCA 28598
                                      4555555666667899************************************************* PP

yeast APRT(L14434.1)            523   tccctagcactggcattgggagttggatttgttccaataaggagagttggaaaattgccggg... 584
                                          t gc  t     t ggag  g  tttgt cc  t ag a a   gg aa  t cc gg
potential L.sordida APRT      28597   ATAATCGCTTTAAGGCTCGGAGCCGCCTTTGTACCTGTCAGAAAACAAGGTAAGCTTCCTGGacc 28533
                                      99999999999998888999****************9999999888888888877776665511l PP

yeast APRT(L14434.1)            585   ...tgagtgtgcttc.............................t.ataaca...........tt 605
                                         tg   gtgc tc                             t  taaca           t
potential L.sordida APRT      28532   atgTGTCGGTGCCTCctacgagaaggaatacggtgtggtacatcTcTTAACAattttttgtacTG 28468
                                      11122222223222211111111111111111110000001111112223211111111111133 PP

yeast APRT(L14434.1)            606   cacaaaatta..........gaccatgaagaaatttttgaaatgcaggttgaagccattcccttt 660
                                      cacaaa tt           g cc   a ga at tttga atgca g   a gc attc
potential L.sordida APRT      28467   CACAAATTTGatttcgttcgGCCCTATAGGACATATTTGAGATGCAAGAAAATGCGATTCAACCA 28403
                                      566665433211112222214455566788999999**********************99998888 PP

yeast APRT(L14434.1)            661   gattccaacgtggttgtcgtggatgatgttctcgc.............................. 695
                                      g     a cgt  t gt  t ga gat tt t gc
potential L.sordida APRT      28402   GGGCAAAGCGTAATAGTTATTGACGATCTTATAGCgacaggtttgtgtaaaaacttccctacatc 28338
                                      88888888888888888888888888877766655511111111111111111111111111111 PP

yeast APRT(L14434.1)            696   ..................caccggtggaacggcatatgctgcgggcgaccttatcagacaagtgg 742
                                      cac ggtgg  c gc   tgctgc ggcga  t  t   a a   gg
potential L.sordida APRT      28337   tggtctaatctaagcaaaCACAGGTGGTTCTGCGCGTGCTGCTGGCGAGTTGGTGGCAAAGCAGG 28273
                                      11112222222222222224556778888888888999999999999999988888777777777788 PP

yeast APRT(L14434.1)            743   gtgctcatattctggaatatgactttgtgctagtgttagatagtctacatggtgaggag.aaatt 806
                                      g g   a a t tggaata   cttt t  t g   t  at  tct ca gg   g a  aa tt
potential L.sordida APRT      28272   GCGGAAAAACTTTGGAATACCTCTTTATCATTGAACT-CATGTTCTTCAGGGCTGGAACcAAGTT 28209
                                      8888888999999999999988888777665555433 3.345555666666665544433213445 5 PP

yeast APRT(L14434.1)            807   atctgctcctattttttc 824
                                      tgctcc    t  tc
potential L.sordida APRT      28208   GGATGCTCCGGCATACTC 28191
                                      555666666555555555 PP
```

Supplementary Figure 1A. The result of nhmmer search against the genomic scaffolds from *L. sordida.* *APRT* sequence in yeast (Genbank ID: L14434.1) was used as a query sequence.

```
C.cinerea APRT, mRNA(XM_001837049.1)      7 gacgtggaattcttgaagagtcggttgagcgcacatatagatttccccagga................... 58
                                            gacgt ga t+c+t aa   +c++ttga+ +  ca++  ga+tt cc a+ga
L.sordida APRT                          28866 GACGTTGAGTACATTAAAGACCAATTGACATTTCACCGTGACTTTCCAAAGAaggtggggcttgtcagaac 28796
                                            6788888888888888887778888888888888888888888888887776611111111111111111111 PP

C.cinerea APRT, mRNA(XM_001837049.1)     59 ............................aggggattgtgtttttggatatttttccaatcttgagagatcc 101
                                                                        aggg at+gt+ttt t ga+at tt+cc  t +t++g gatcc
L.sordida APRT                          28795 tggtagattctaaacttatcccaatgtcAGGGCATCGTCTTTCTTGACATATTCCCTCTTCTCCGTGATCC 28725
                                            1111111222222222222222222222467889************************************* PP

C.cinerea APRT, mRNA(XM_001837049.1)    102 tgtcgtgttcgagacgcttatctcgcatatcgttcagcacgtcgcatcgcacactatcgcaatttctctct 172
                                            t tc + tt ga ac ct+atc+cgcat t gt+ca+ca gt +catc  a actatc c+a+ tc+c+c+
L.sordida APRT                          28724 TATCACTTTTGAAACACTCATCACGCATTTTGTCCACCATGTAACATCATATACTATCACCAAGTCACCCA 28654
                                            *********************************************************************** PP

C.cinerea APRT, mRNA(XM_001837049.1)    173 caaagaagattgatgtggtggtaggtttggatgctcggggcttttttttgggtccaattattgcgctgagg 243
                                            c a +aagat gatgt++t+gt ggt+t ga+gc cg gg ttt+t  t ggtccaat at+gc  t agg
L.sordida APRT                          28653 CGAGAAAGATCGATGTTATCGTTGGTCTTGACGCACGCGGTTTTCTCCTCGGTCCAATAATCGCTTTAAGG 28583
                                            *********************************************************************** PP

C.cinerea APRT, mRNA(XM_001837049.1)    244 cttggtgctgcttttgtgccagttaggaagaagggcaagctccctggagaatgtgtttctgttacatatga 314
                                            ct gg gc+gc tttgt cc gt+ag aa  a gg aagct cctgga  atgtgt   tg+  c+ta+ga
L.sordida APRT                          28582 CTCGGAGCCGCCTTTGTACCTGTCAGAAAACAAGGTAAGCTTCCTGGACCATGTGTCGGTGCCTCCTACGA 28512
                                            *********************************************************************** PP

C.cinerea APRT, mRNA(XM_001837049.1)    315 gaaggaatatggag...........................c............................. 329
                                            gaaggaata gg g                           c
L.sordida APRT                          28511 GAAGGAATACGGTGtggtacatctcttaacaattttttgtaCtgcacaaatttgatttcgttcggccctat 28441
                                            *********998761111111111111111111111111110111122222111111111111111111222 PP

C.cinerea APRT, mRNA(XM_001837049.1)    330 .ggattcatttgaaatgcaggcggatgctatcaagtcaggtcagtctgtagttatcgtagatgacttgatt 399
                                             gga+  atttga atgca+g   atgc at  a +cagg ca++  gta+t  t +t ga+ga +t+at
L.sordida APRT                          28440 aGGACATATTTGAGATGCAAGAAAATGCGATTCAACCAGGGCAAAGCGTAATAGTTATTGACGATCTTATA 28370
                                            23455567899***********************************************************99 PP

C.cinerea APRT, mRNA(XM_001837049.1)    400 gc...........................a.....................actggtggttctgcgaaggc 422
                                            gc                           a                     ac+ggtggttctgcg   gc
L.sordida APRT                          28369 GCgacaggtttgtgtaaaaacttccctacAtctggtctaatctaagcaaacACAGGTGGTTCTGCGCGTGC 28299
                                            98222222222222222221111111111011111122222222222222235678 9************** PP

C.cinerea APRT, mRNA(XM_001837049.1)    423 cgcaggcgagctagtcgagaaactgggaggcaagactctagagtacattttcattatagagttacttttct 493
                                             gc ggcgag t gt g+ aa c+ggg gg aa+act t ga+tac+t+tt at+at ga++t+ t ttct
L.sordida APRT                          28298 TGCTGGCGAGTTGGTGGCAAAGCAGGGCGGAAAAACTTTGGAATACCTCTTTATCATTGAACTCATGTTCT 28228
                                            *********************************************************************** PP

C.cinerea APRT, mRNA(XM_001837049.1)    494 tgaaggccgcagagaagctcgacgctcccgtctattctattgtccaatgcgaagactg 551
                                            t+a ggc g a ++aag t ga gctcc g  ta tc at+gt caat+ ga ga tg
L.sordida APRT                          28227 TCAGGGCTGGAACCAAGTTGGATGCTCCGGCATACTCGATCGTTCAATCGGATGATTG 28170
                                            ****************************************************999876 PP
```

Supplementary Figure 1B. Predicted coding region of *L. sordida* APRT. Coding region of L. sordida APRT was predicted using nhmmer search. APRT mRNA sequence in *C.cinerea* (Genbank ID: XM_001837049.1) was used as a query sequence.

```
ATGGACGTTGAGTACATTAAAGACCAATTGACATTTCACCGTGACTTTCCAAAGAAGgtggggcttgtcag
M  D  V  E  Y  I  K  D  Q  L  T  F  H  R  D  F  P  K  K

aactggtagattctaaacttatcccaatgtcagGGCATCGTCTTTCTTGACATATTCCCTCTTCTCCGTGA
                                 G  I  V  F  L  D  I  F  P  L  L  R  D

TCCTATCACTTTTGAAACACTCATCACGCATTTTGTCCACCATGTAACATCATATACTATCACCAAGTCAC
 P  I  T  F  E  T  L  I  T  H  F  V  H  H  V  T  S  Y  T  I  T  K  S  P

 CCACGAGAAAGATCGATGTTATCGTTGGTCTTGACGCACGCGGTTTTCTCCTCGGTCCAATAATCGCTTTA
  T  R  K  I  D  V  I  V  G  L  D  A  R  G  F  L  L  G  P  I  I  A  L

AGGCTCGGAGCCGCCTTTGTACCTGTCAGAAAACAAGGTAAGCTTCCTGGACCATGTGTCGGTGCCTCCTA
R  L  G  A  A  F  V  P  V  R  K  Q  G  K  L  P  G  P  C  V  G  A  S  Y

  CGAGAAGGAATACGGTGTGgtacatctcttaacaatttttgtactgcacaaatttgatttcgttcggccc
 E  K  E  Y  G  V

tatagGACATATTTGAGATGCAAGAAAATGCGATTCAACCAGGGCAAAGCGTAATAGTTATTGACGATCTT
     D  I  F  E  M  Q  E  N  A  I  Q  P  G  Q  S  V  I  V  I  D  D  L

ATAGCGACAGgtttgtgtaaaaacttccctacatctggtctaatctaagcaaacacagGTGGTTCTGCGCG
I  A  T  G                                                 G  S  A  R

TGCTGCTGGCGAGTTGGTGGCAAAGCAGGGCGGAAAAACTTTGGAATACCTCTTTATCATTGAACTCATGT
 A  A  G  E  L  V  A  K  Q  G  G  K  T  L  E  Y  L  F  I  I  E  L  M  F

TCTTCAGGGCTGGAACCAAGTTGGATGCTCCGGCATACTCGATCGTTCAATCGGATGATTGA
  F  R  A  G  T  K  L  D  A  P  A  Y  S  I  V  Q  S  D  D  *
```

Supplementary Figure 2. The genomic DNA and the deduced amino acid sequence of *L. sordida APRT* gene. Upper case ; exon sequences, lower case ; intron sequences ; bold upper case ; predicted amino acid sequence. Note that the underlines of the introns display GT-AG consensus for splicing sites.

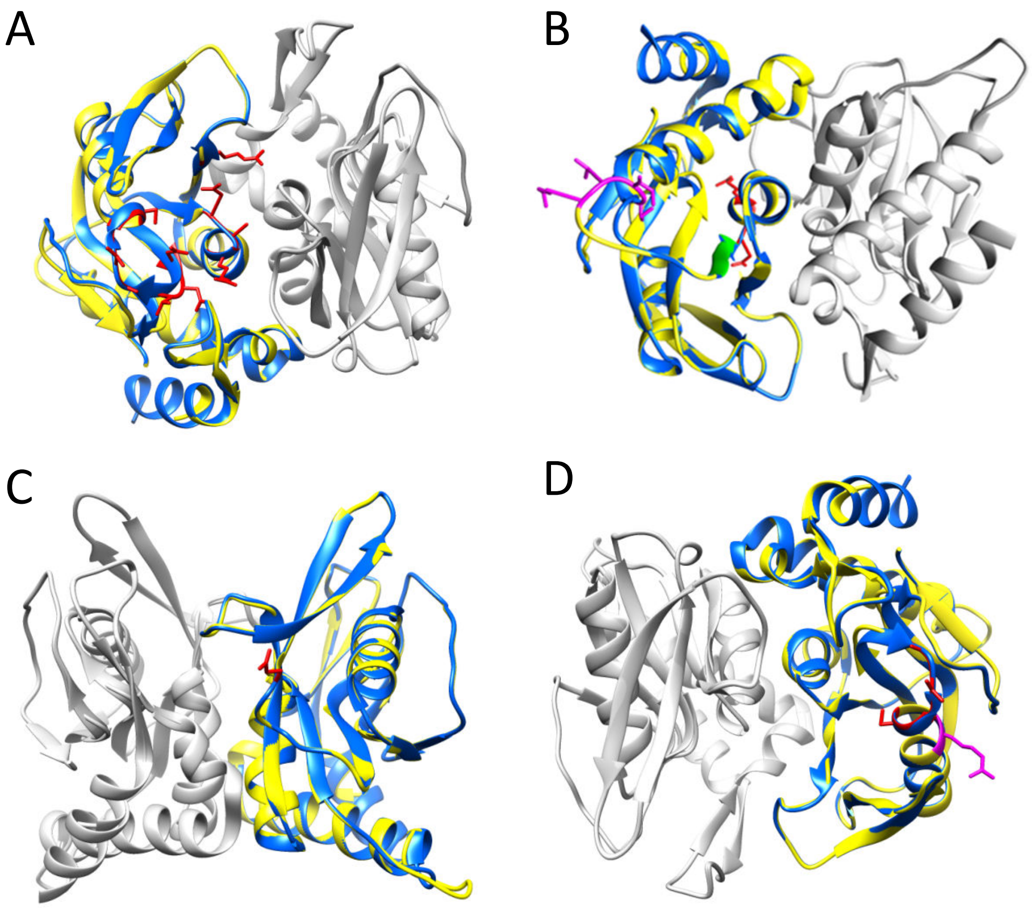

Supplementary Figure 3. Structures of APRTs in *L. sordida* and *S. cerevisiae*. Three-dimensional structural modeling was carried out on SwissModel server with the *S. cerevisiae* (Protein data bank code, 1G2Q) as the template. Ribbon diagram shows the overlap of the *S. cerevisiae* APRT monomer (blue) and *L. sordida* APRT monomer (yellow). (A) Putative phosphoribosyl pyrophosphate (PRPP)-binding motifs and 5'-phosphate contact region of *L. sordida*. The conserved amino acid residues related in the PRPP and 5'-phosphate binding are shown in red. (B) The missing region in *L. sordida* APRT. A missing region (51-Thr-Ile-Thr-Lys-54), citrate contact region (67-Asp-Ala-Arg-69) and alfa-helix (62-Val-Ile-Val-Gly-Leu-66) near 5' citrate contact region are shown in magenta, red and green, respectively. (C) Glu-116 positioned on the dimer surface. Glu-116 conserved between *L. sordida* and *P. involutes* is shown in red. (D) Arg-139 is component of a-helix near 5' phosphate binding motif. Arg-139 and 5' phosphate binding motif (133-Ala-Thr-Gly-Gly-Ser-137) are shown in magenta and red, respectively.

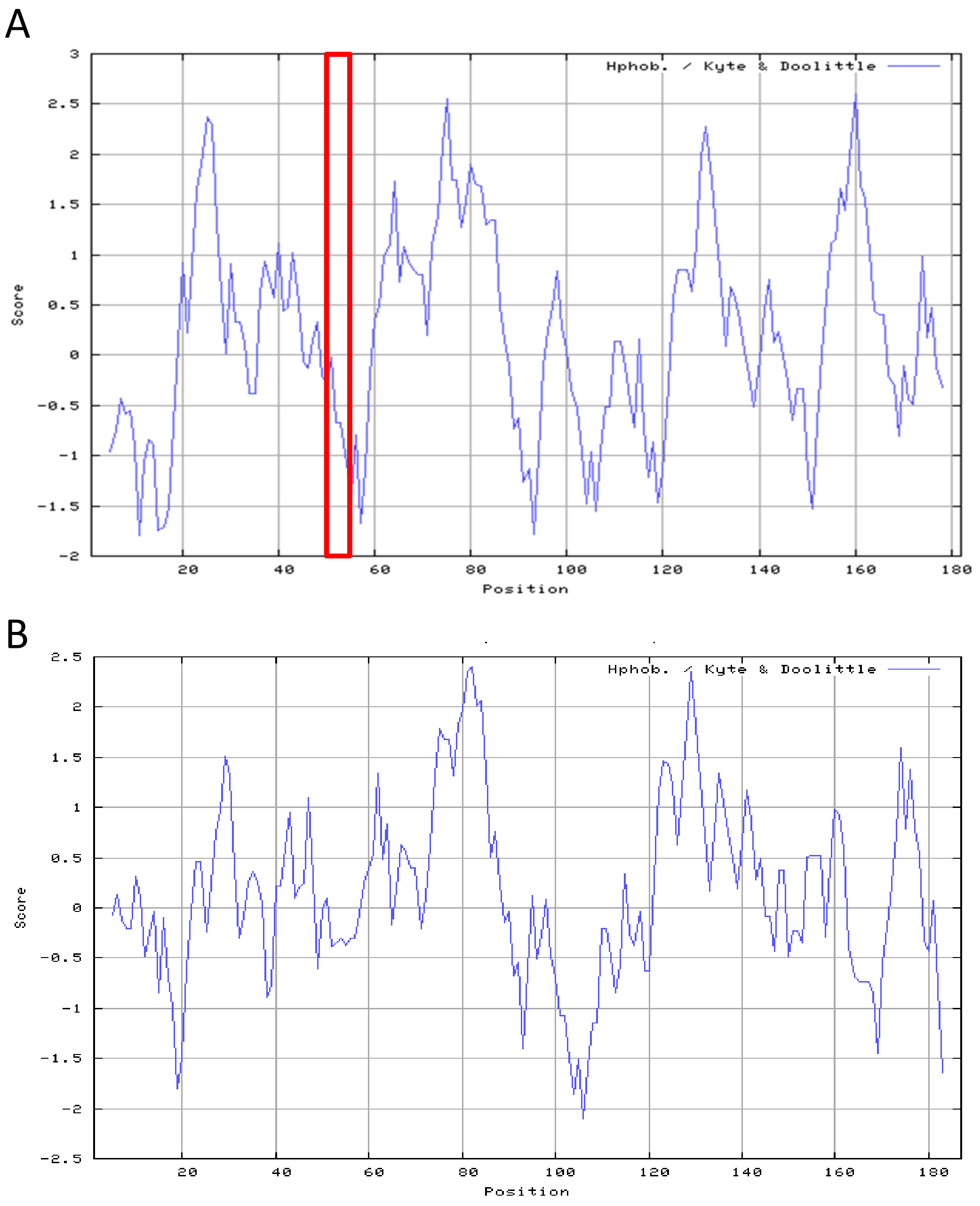

Supplementary Figure 4. Hydropathy Plot for *L. sordida* APRT(A) and *S. cerevisiae* APRT (B) (Protein data bank code, 1G2Q). Hydropathy plot was created using the Protscale tool (http://web.expasy.org/protscale/). The missing region in *L. sordida* APRT is boxed in red.
